# Solitary hepatic lymphangioma: a one-case report

**DOI:** 10.1186/2193-1801-3-314

**Published:** 2014-06-25

**Authors:** Qu Liu, Cheng-Jun Sui, Bao-Shan Li, Ang Gao, Jian-yue Lu, Jia-Mei Yang

**Affiliations:** Department of Special Treatment, Eastern Hepatobiliary Surgery Hospital, Second Military Medical University, Shanghai, 200438 China; Department of Hepatobiliary Surgery, People’s Republic of China No. 254 Hospital, Tianjin, 300142 China

**Keywords:** Solitary cyst, Hepatic lymphangioma, Surgical treatment

## Abstract

Hepatic lymphangiomas, malformations of the liver lymphatic system, are extremely rare conditions in adults. A 41-year-old man presented with right upper abdominal pain for 6 months was introduced in this report. Ultrasound (US) and computed tomography (CT) scan demonstrated a giant cystictumor with a pedunculatedextrahepatic growth pattern. Due to diagnostic uncertainty, a partial hepatectomy was performed and pathological results confirmed the diagnosis of solitary hepatic lymphangioma. In this article, we reviewed the clinical and pathology features, preoperative diagnostic challenges, and treatments of hepaticlymphangiomas.

## Background

Lymphangiomas are benign neoplasms regarded as congenital malformations of the lymphatic system (Stavropoulos et al. 1994). Most lymphangiomas are located in the head, neck, and axilla, where the loose connective tissue allows for easy expansion of lymphatic channels. Intraabdominal cases account for less than 5% of all lymphangiomas (Losanoff et al. 2003). Hepatic Lymphangiomas are characterized by cystic dilatation of the lymphatic vessels in the hepatic parenchyma usually observed in children and adolescents (Bertino et al. 2014; Asch et al. 1974; Zhang et al. 2013). In most cases, the hepatic lesion is usually part of multi-organ involvement including the spleen, kidney, skeleton, gastrointestinal tract, mesentery, lung, pleura, pericardium and other tissues (Stavropoulos et al. 1994; Koh & Sheu 2000). A solitary hepatic lymphangioma in an adult is extremely rare and lack of specific clinical symptoms, therefore, it is easy to be misdiagnosed. The aim of this report is to introducing the experience in managing an adult with a giant liver cystic lymphangioma.

### Case report

A 41-year-old male with a 6-month history of left upper abdominal pain visited our department. A large, palpable soft mass 8 cm below the left costal margin was found.

**Figure 1:**
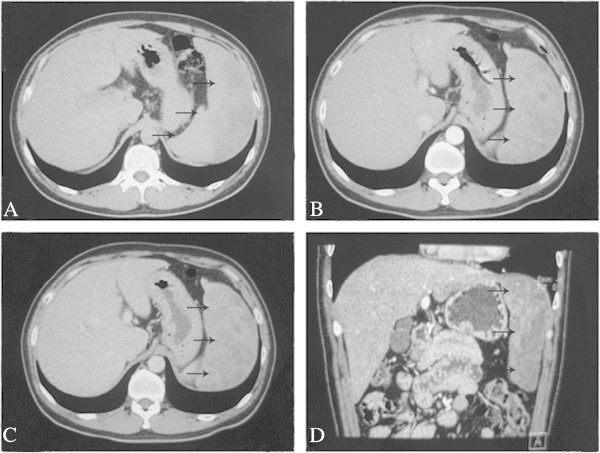
**Contrast-enhanced CT scan shows heterogeneous enhancement of the giant cystic lesion. A)** the plain CT scan, **B)** the arterial phase of CT, **C)** the portal venous phase of CT, **D)** CT scan in the sagittal plane. Arrows indicate the hepatic lesion.

Hematology and biochemistry results were normal while Hepatitis B surface antibody (HBsAb) and Hepatitis B core antibody (HBcAb) were positive. Results of kidney function, electrolytes, and all tumor markers related to the liver were negative. Brain computed tomography (CT) scan and chest X-ray did not find other lesions in the patient.Abdominal ultrasound (US) showed a giant mixed-echoic mass (15 cm × 10 cm) with a pedunculatedextrahepatic growth pattern. An enhanced abdominal CT scan confirmed a giant cystic hepatic lesion with no capsule. The lesion was demonstrated with a heterogeneous enhancement in the arterial phase (Figure 1). The laboratory and image findings were not sufficient to differentiate the benign or malignant nature of the mass.

**Figure 2:**
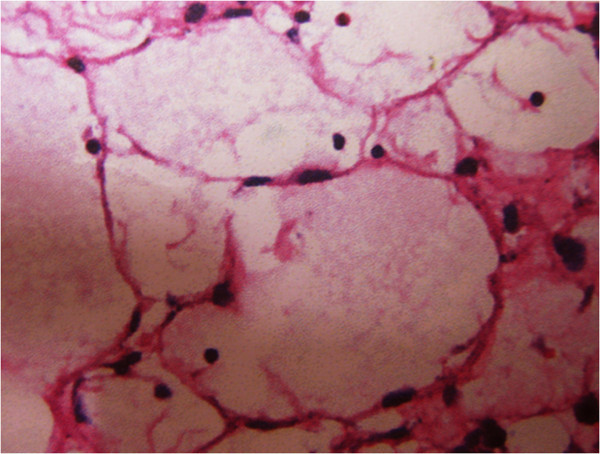
**Microscopically, the lesion is composed of anastomosing lymphatic spaces lined by attenuated endothelial-like cells with mature differentiation and containing homogeneous pink fluid.**

At laparotomy, a huge, smooth tumor was found in segment II with pedunculated extrahepatic growth, with no other pathology found in the abdomen. The tumor was completely removed bylocal resectionof segment II of the liver using an ultrasonic scalpel and cavitron ultrasonic surgical aspirator after occlusion of the left hemihepatic vascular.Histology of the resected specimen revealed a giant cystic mass about 16.2 cm × 13 cm × 4.3cmm in size. On macroscopic examination, the tumor was cystic and multilocular. The cyst had a thick, gray-white wall and was filled with serous fluid containing a small amount of blood. Microscopically, the tumor was composed of irregular and expanded lymphatic vessels, lined by flattened endothelial cells (Figure 2). Immunohistochemical study was carried out on formalin-fixed, paraffin-embedded tissues. The endothelial-like cells were almost all positive for CK18, and pCEA, while Hep-1, HBsAg, CK19, CD34, HBcAg, β-catenin, MUC-1, MAT1, GLy-3, and KIAA were all negative. The final histopathologic diagnosis for the specimen was solitary hepatic lymphangioma.

The postoperative course was uneventful and the patient has been followed up for 30 months. Now, he is symptom-free with no evidence of recurrence on subsequent abdominal imaging.

## Discussion

Lymphangiomas are a group of benign tumors which are composed of lymphatic spaces lined by attenuated endothelium. Histologically, lymphangiomascan be divided into three groups: capillary, cavernous, and cystic lymphangioma (Asch et al. 1974). The exact pathogenesis of lymphangioma remains largely unknown, although the congenital developmental abnormality of the lymphatic tissue, dilatation of abnormal channels, and localized lymphatic obstruction are thought to be the important causes (Asch et al. 1974; Enzinger & Weiss 1995). Other pathogeneses discussed include trauma, inflammatory and fibrotic processes, as well as vascular endothelial permeability disorders. Lymphangiomas are usually found in the neck, axilla, mediastinum, retroperitoneal, soft tissues or other areas rich in lymphoid tissue (Losanoff et al. 2003). Abdominal lymphangiomas, especially solitary ones in one lobe of the liver are extremely rare (Losanoff et al. 2003; Bertino et al. 2014; Koh & Sheu 2000). Hepatic lymphangiomas are characterized by cystic dilatation of the lymphatic vessels in the hepatic parenchyma (Zhang et al. 2013; Conlon et al. 1996) filled withlymph fluid (Enzinger & Weiss 1988).

Hepatic lymphangiomas usually have non-specific clinical signs or symptoms (Matsumoto et al. 2010; Allen et al. 2006). In our case, the main complaint of patient was left upper abdominal pain, which may be related to the expansion of the giant lesion compressing the surrounding structures. Laboratory and Imaging results were of limited use for the preoperative diagnosis of lymphangioma. Because all tumor markers related to the liver were negative, the possibility of hepatocellualar carcinoma and intrahepatic cholangiocarcinoma was relative low (Malaguarnera et al. 2013; Biondi et al. 2012; Bertino et al. 2012). On US, CT, and Magnetic Resonance (MRI), hepatic lymphangioma may appear as a cystic or multi-cystic mass with internal septations, and it is difficult to differentiate it from other cystic disease such as bile duct cyst, biliary cystadenoma and cystadenocarcinoma, or hepatic hydatidosis (Levy et al. 2004), despite the fact that MRI is very helpful in differentiating lymphangioma from a true solid tumor (Siegel et al. 1989). In the patient, the lack of specificity of these preoperative investigations and the rarity of hepatic lymphangioma made it very challengeable to give the accurate preoperative diagnosis. Pathological examinations have been suggested as the only way to identify the nature of the hepatic mass (Steenbergen et al. 1985). We did not perform percutaneous biopsy considering the low positive rate, the risk of bleeding and malignant seeding. The treatment for hepatic lymphangioma is complete resection. Incomplete removal often leads to the recurrence of the cyst (Roisman et al. 1989). Resection is often required for symptom control and accurate diagnosis. Patients with giant hepatic lymphangiomas that are not resectable or with severe impairment of liver function can be treated by orthotopic liver transplantation (Tepetes et al. 1995). The prognoses following surgical treatment are excellent. Alternative therapies for patients who are not suitable for surgical treatment include injection of ethanol or OK-432 (Banieghbal et al. 2003) directly into the lymphangiomas (Steenbergen et al. 1985), but an exact diagnosis is not possible in this way and the psychological impact of the tumor would not be eliminated. In practice, physicians should follow the principles of evidence-based medicine and combine the patient’s wishes with the previous treatment experience to choose the optimal treatment.

## Conclusions

In conclusion, we report a rare case of huge hepatic lymphangioma with pedunculatedextrahepatic growth in a 41-year-old male. Preoperative examinations were not sufficient to differentiate the nature of the tumor. The total resection of the tumor resolved all his symptoms, and the pathological finding revealed the true nature of the mass. The long-term prognosis following surgery is quite satisfactory.

### Consent statement

Written informed consent was obtained from the patient for publication of this case report and any accompanying images. A copy of the written consent is available for review by the Editor of this journal.

## Authors' information

The corresponding author is the deputy head of the Society of hepatobiliary surgery, Branch Association of surgery, China Medical Association. Eastern Hepatobiliary Surgery Hospital is the biggest therapeutic centre of hepatobiliary surgery in China.

## Abbreviations

HBsAb: Hepatitis B surface antibody
HBcAb: Hepatitis B core antibody
CT: Computed tomography
MRI: Magnetic resonance.
